# Prise de remèdes de phytothérapie et implications opératoires: à propos d'un cas

**DOI:** 10.11604/pamj.2014.17.268.2687

**Published:** 2014-04-12

**Authors:** Firas Dhouib, Mohammed Frikha, Walid Zineddine

**Affiliations:** 1 Service d'Anesthésie Réanimation Chirurgicale, CHU Habib Bourguiba, Sfax, Tunisie

**Keywords:** Phytothérapie, remèdes, anesthésie, complications, Phytotherapy, remedies, anesthesia, complications

## Abstract

Les interactions médicamenteuses sont nombreuses et doivent faire l'objet d'une attention particulière à la consultation préanesthésique. Certains médicaments ne sont pas toujours spontanément révélés au médecin anesthésiste. Il s'agit le plus souvent de thérapeutiques dites “non traditionnelles” telle que la phytothérapie, méthode thérapeutique qui utilise l'action des plantes médicinales. On rapporte le cas d'un homme âgé de 38 ans, proposé pour ostéosynthèse d'une fracture des corps vertébraux de D11 et D12, ayant présenté un saignement per opératoire inhabituel rapporté à une consommation de grandes quantités d'ail.

## Introduction

Lors de la consultation préanesthésique, l'arrêt ou la poursuite de certains médicaments peut être envisagés en prévision d'actes chirurgicaux ou d'investigations réalisés sous anesthésie. Le médecin anesthésiste a la tâche parfois difficile de veiller à dépister les interactions possibles d'un traitement médicamenteux pris au long cours, et surtout de prendre en compte celles pouvant survenir au cours de la période périopératoire [1].

La tâche est encore plus difficile lorsque certains traitements ne sont pas spontanément révélés par le patient et demeurent ignorés par le médecin consultant. Ce phénomène est fréquemment observé avec la phytothérapie qui bénéficie d'un véritable engouement en Europe et aux États-Unis [2, 3], où les différentes études estiment en effet que 5 à 20% des patients sont consommateurs. Même si elle est rare, la morbi-mortalité associée à cette thérapeutique est réelle. Nous essayons de montrer, par le rapport d'une observation, l'importance d'interroger les patients à la consultation d'anesthésie sur une éventuelle prise de remèdes de phytothérapie dans la prévention d'incidents parfois graves.

## Patient et observation

Il s'agit d'un homme âgé de 38 ans, sans antécédents pathologiques connues, victime d'un accident de travail occasionnant un traumatisme thoracique avec des fractures de deux côtes sans épanchement, associé et un traumatisme dorsal occasionnant une fracture des corps vertébraux de D11 et D12 sans atteinte neurologique associée. Une ostéosynthèse chirurgicale a été indiquée.

A l'examen préopératoire, le patient a affirmé ne pas prendre de médicaments en particulier des antiagrégants plaquettaires. Le bilan préanesthésique était correct avec une hémoglobine à 12.4, des plaquettes à 235000, un taux de prothrombine à 76% et un temps de céphaline activée à 32/30.

En peropératoire, le temps de dissection a été associé à un saignement diffus au champ opératoire gênant le chirurgien. Ce saignement estimé à 2500 ml, a été jugé anormal puisque le saignement per opératoire habituel dans la chirurgie du rachis traumatique dans notre équipe est de 200 ± 50 ml par vertèbre fixée. Pour notre patient, cinq vertèbres ont dû être fixées et le saignement théorique ne devrait pas dépasser les 1250 ml.

Les circonstances du saignement étaient habituelles: la pression abdominale était correcte au alentour de 20 mmHg, la technique chirurgicale était habituelle, le chirurgien était expérimenté et le patient n’était pas en hypothermie. Le chirurgien n'avait pas d'explication chirurgicale de ce problème d'hémostase. Le saignement diffus a persisté malgré la baisse de la tension artérielle. Une dose de charge d'acide tranexamique de 10 mg.kg^-1^ suivie d'une perfusion continue de 1 mg.kg^-1^.h^-1^ ont été administrées au patient, et une transfusion de 2 concentrés globulaires et de 4 plasmas frais congelés a été nécessaire en per opératoire.

Les suites opératoires étaient simples, le patient a été extubé 2 heures après la fin de l'intervention. Le bilan biologique après transfusion a montré une hémoglobine à 9,2 g/dl, des plaquettes à 163000/mm^3^, un taux de prothrombine à 72% et un fibrinogène à 3. Aucun déficit neurologique n'a été observé. Des nouveaux tests de la coagulation avec recherche de maladie de Willebrand, d'hémophilie et de déficit en facteur XI n'ont pas objectivé d'anomalies. La recherche d′un anticoagulant circulant de type auto-anticorps anti-facteur VIII ou anti-facteur IX n'a pu être réalisée par manque de réactif. Cependant le temps de saignement (Ivy incision) était anormalement allongé à 16 min.

Un entretien plus fouillé avec le patient 24 heures après a permis de dévoiler une habitude de prise quotidienne d'ail par le patient à des quantités importantes. Cette habitude a été adoptée et transmise de génération en génération par tous les membres de sa famille pour prévention d'une hypercholestérolémie familiale. Cette phytothérapie avec de l'ail qui est connue pour avoir une activité inhibitrice de l'agrégation plaquettaire de manière dose dépendante, a été retenue comme cause du saignement observé en per opératoire.

## Discussion

Certains médicaments ne sont pas toujours spontanément révélés à l'anesthésiste. Il s'agit le plus souvent de thérapeutiques dites «non traditionnelles ». Parmi celles-ci la phytothérapie en est un exemple. La phytothérapie, étymologiquement le traitement par les plantes, est une méthode thérapeutique qui utilise l′action des plantes médicinales. Elle est probablement la médecine la plus ancienne.

Trois études récentes [2, 3, 4] confirment l′enthousiasme actuel pour la phytothérapie. Aux États-Unis, la consommation de plantes a augmenté d′environ 400% en 10 ans. En France, le marché affiche une croissance de 14%.

L′analyse de la littérature a permis d′identifier huit produits susceptibles d′interférer notablement avec la période péri opératoire [5]. Parmi eux, l'ail contribue à la prévention de l′athérosclérose en réduisant la pression artérielle et les taux sanguins des lipides et du cholestérol [5]. Un des composants de l′ail, l′ajoene, inhibe l′agrégation plaquettaire de façon dose dépendante et de manière irréversible. L'ail a de tels effets en doses journalières d'environ 800 mg par jour prises plus que 2 fois par semaine. La littérature disponible sur la signification clinique correspondante est composée essentiellement de rapports de cas, et non de revues systématiques. Un risque accru d'hémorragies a été décrit chez les patients ayant pris des comprimés ou capsules d'extraits d'ail [6, 7]. Rose et al. rapportent un cas d′hématome médullaire spontané attribué à une consommation excessive de cette plante [8].

Des études européennes ont montré que jusqu′à 50% des patients prennent des remèdes à base de plantes avant une opération [2, 9], et n'en parlent généralement que lors d'une anamnèse ciblée. Dans le travail de Skinner et al [2], les patients ne jugeaient pas utile de porter à la connaissance de l'anesthésiste la prise de phytothérapie.

Il est nécessaire en consultation d′anesthésie, lors de l′anamnèse, de rechercher l′adhésion du patient à une phytothérapie car elle n′est pas spontanément révélée [2]. Même si elle est rare, la morbi-mortalité associée à cette thérapeutique est réelle. La société française d'anesthésie et réanimation a publié des recommandations à ce sujet [10]. Ainsi, il est recommandé de questionner les patients de façon très ciblée sur une prise éventuelle de remèdes phytothérapeutiques, étant donné que les patients tendent souvent à ne pas mentionner ces médicaments spontanément.

Lorsque l'on souhaite éviter tout effet antiagrégant plaquettaire de l'ail, une interruption de 10 jours avant l′intervention permettant un renouvellement plaquettaire.

## Conclusion

Tout candidat à une anesthésie est invité à signaler les extraits de plantes qu'il prend lors de la consultation pré anesthésique. Actuellement et à tort, cette information est rarement demandée par le médecin anesthésiste, et encore plus rarement rapportée spontanément par le malade, estimant que la phytothérapie est une médecine douce sans aucun effet secondaire.
